# Modified staging classification for intrahepatic cholangiocarcinoma based on the sixth and seventh editions of the AJCC/UICC TNM staging systems

**DOI:** 10.1097/MD.0000000000007891

**Published:** 2017-08-25

**Authors:** Ze-Wu Meng, Wei Pan, Hai-Jie Hong, Jiang-zhi Chen, Yan-Ling Chen

**Affiliations:** a Department of Hepatobiliary Surgery, Fujian Medical University Union Hospital; b Key Laboratory of Ministry of Education for Gastrointestinal Cancer, Fujian Medical University, Fuzhou, Fujian, People's Republic of China.

**Keywords:** clinicopathological characteristics, intrahepatic cholangiocarcinoma, prognosis, TNM staging

## Abstract

Intrahepatic cholangiocarcinoma (ICC) was differentiated from hepatocellular carcinoma, as defined in the American Joint Committee on Cancer (AJCC) 6th edition staging manual, using the revised staging system described in the AJCC 7th edition staging manual. This study was conducted to analyze the application of the AJCC 6th and 7th edition staging classifications and to evaluate a modified staging classification to potentially reduce the limitations associated with the different AJCC staging systems.

We compared the prognostic value of cancer staging using data from the Surveillance, Epidemiology, and End Results database (N = 2124). The Kaplan–Meier method and Cox regression models were used to analyze survival. The Harrell concordance index (C-index) was used to analyze the discriminative abilities of cancer staging.

Patients with stages I and II disease were found to have similar prognoses according to the 6th edition staging system. Using the 7th edition staging system, a low proportion of patients had stage III disease (5.0%), and the hazard ratio (HR) for stage III disease was comparable to that of stage IV disease (stage III and IV, 2.653 and 2.694). We modified the AJCC staging classification by adopting the 7th edition T, N, and M definitions and the 6th edition staging definitions. Consequently, the proportion of patients with stage III disease increased (22.8%). The HR for stage IV disease was higher than that for stage III disease (stage III and IV, 2.425 and 2.956). Meanwhile, the C-index of the modified AJCC staging system was 0.721 (95% CI: 0.696–0.745), which was significantly higher than the AJCC 7th edition staging system (0.694, *P* < .001), and the AJCC 6th edition staging system (0.712, *P* = .033). Moreover, in the stratified data, the differences between the stages identified using the modified AJCC staging classification were significant, especially among patients over 60 years in age, white patients and patients who underwent surgery.

These findings suggest that the modified AJCC staging classification may be applicable to the staging of ICC and can be adopted in clinical practice.

## 1 Introduction

The incidence rate of intrahepatic cholangiocarcinoma (ICC) has increased over the past several decades, both in the USA and worldwide.^[1]^ ICC accounts for approximately 5% to 30% of all primary liver cancer cases.^[2–4]^ As the second most common liver cancer, ICC is highly malignant and has an extremely poor prognosis.^[5]^ Therefore, an accurate and simple staging system is needed to provide prognostic information and stratify patients by risk, as these variables are factors of primary importance in the determination of therapeutic methods and assessment of prognosis.

In the 6th edition staging system for hepatic malignancies in the American Joint Committee on Cancer (AJCC)/Union for International Cancer Control (UICC) manual, the staging of ICC is identical to that of hepatocellular carcinoma (HCC) and is based on a tumor-node-metastasis (TNM) staging system created using data from HCC patients.^[6]^ Because ICC has different carcinogenic mechanisms and biological behavior from HCC, it may not be appropriate to use the same staging classification, as this classification system is mostly based on HCC data.^[7]^ Therefore, ICC was separated from HCC in the revised staging system included in the AJCC 7th edition staging manual,^[8]^ which is mainly derived from research conducted by Nathan et al.^[9]^ This represents the first time that ICC has an independent staging system in the AJCC staging manual. The independent staging system for ICC focuses on the presence of multiple tumors, lymph node metastasis, and vascular invasion, each of which was included based on data reported by Nathan et al.^[9]^ The differences between the AJCC 6th and 7th edition staging manuals are described in Table 1. The staging system included in the AJCC 7th edition is less complex than that included in the AJCC 6th edition,^[10]^ but whether the former is a significantly better tool for evaluating ICC patients than the latter has not yet been determined. Few studies have been conducted to validate the AJCC 7th edition staging system or to provide suggestions for revision. Jiang et al^[11]^ after assessing the prognostic validity of the system using data from 344 patients with ICC who underwent liver resection, designed the Fudan score and provided evidence that, based on clinical factors, this score better predicts the prognosis of ICC patients than the AJCC 7th edition system. Farges et al^[12]^ investigated survival in 163 ICC patients with R0 resection and found that the AJCC 7th edition staging system could accurately predict survival in ICC patients, suggesting that this staging system may be used for ICC patients in clinical settings worldwide. Li et al^[13]^ retrospectively investigated the effectiveness of the revised staging system using data from 283 ICC patients with R0 liver resection, and the results suggested that the revised system may be effective in predicting survival among ICC patients after R0 resection. However, using data from 126 patients with ICC who underwent surgery, Ali et al^[14]^ found that the AJCC 7th edition staging system did not accurately predict the survival of ICC patients. Notably, the accuracy of this staging system in predicting the survival of patients with ICC may be improved by simultaneously assessing tumor size and differentiation. All of the above-described studies focused on ICC patients who underwent surgery and did not comprehensively assess risk across all populations of ICC patients using the AJCC 7th edition staging system due to the use of small sample sizes. In addition, the AJCC 6th edition staging system was not evaluated in these studies, and the possible advantages of this system were not considered.

**Table 1:**
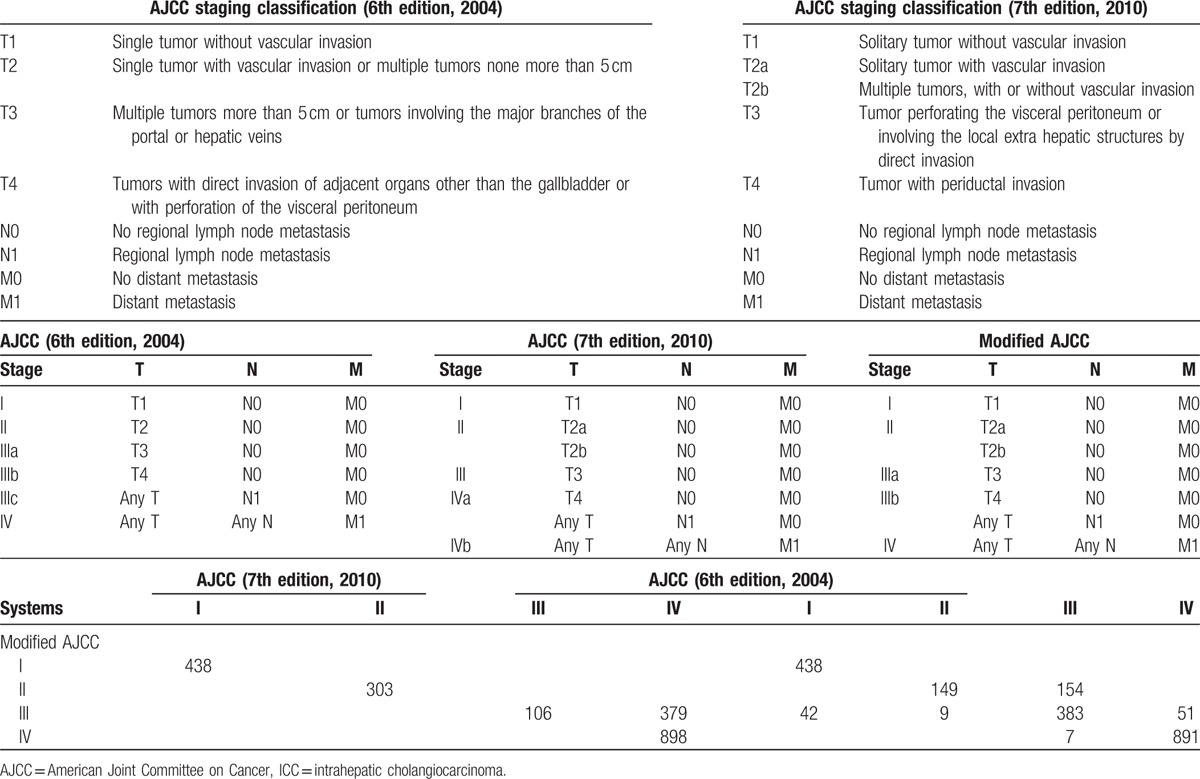
Cross-tabulation of the distribution of patients meeting the different AJCC staging definitions for intrahepatic cholangiocarcinoma using the AJCC 6th edition (2004), AJCC 7th edition (2010), and modified AJCC systems.

**Figure 1:**
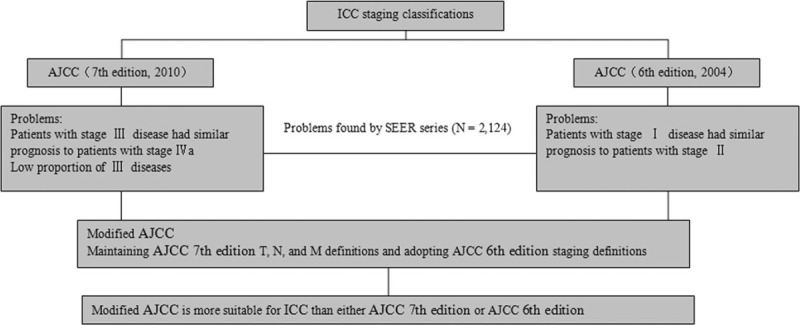
Consort diagram showing the design of the study. Problems in the American Joint Committee on Cancer (AJCC) 6th and 7th edition staging systems were found, and a modified AJCC staging system was proposed using the SEER series. AJCC = American Joint Committee on Cancer, SEER = Surveillance, Epidemiology, and End Results.

The present study was conducted to analyze the application of the AJCC 6th and 7th edition staging classifications using a large data set and to evaluate a modified staging classification to potentially reduce the limitations associated with the different AJCC staging systems (Table 1, Fig. 1). The proposed classification system maintained the T, N, and M definitions of the 7th edition of the AJCC staging system and adopted the AJCC 6th edition staging definitions. The purpose of this study was to assess the prognostic value of the AJCC 6th and 7th edition staging systems in comparison with the prognostic value of the modified AJCC staging system using data from ICC patients stratified according to independent prognostic factors.

## 2 Materials and methods

### 2.1 Patients

The Surveillance, Epidemiology, and End Results (SEER)^[15]^ database (1973 to 2013) was used to identify ICC patients. Patients evaluated between 2010 and 2013 were chosen because both the 6th and 7th editions of the AJCC staging system were used to characterize the patients examined during this time period. Patients were identified based on the International Classification of Diseases for Oncology (3rd edition).^[16]^ The following coding was used: the primary site code for the liver (22.0); the histology code for cholangiocarcinoma (8160); the primary site code for the intrahepatic bile duct (22.1); the histology codes for malignant neoplasm (8000), malignant tumor cells (8001), carcinoma (8010), undifferentiated carcinoma (8020), adenocarcinoma (8140), and cholangiocarcinoma (8160); and a behavior code (3-malignant tumor). TNM information was retrieved based on the following codes: derived AJCC stage group (7th edition; 2010+) and derived AJCC stage group (6th edition; 2004+). The SEER 8.3.2 registry research database was utilized to generate a listing of ICC cases, and the following variables were extracted for the 2376 eligible patients: site recode (intrahepatic bile duct), behavior recode for analysis (malignant), age, race, sex, marital status at diagnosis, year of diagnosis (2010–2013), histological grade, AJCC stage (6th edition, 2004), AJCC stage (7th edition, 2010), tumor stage (6th edition, 2004), tumor stage (7th edition, 2010), node stage, surgical status (yes, no), adjuvant radiotherapy status (yes, no), SEER cause-specific death classification, and survival (months). We excluded patients with SEER cause-specific deaths not classified as first tumors and with comorbidities. Finally, 2124 cases were enrolled in our study. Age at diagnosis was classified as ≤60 years and >60 years. Race was recoded as white, black, or other (includes Asian/Pacific Islander and American Indian/Alaskan native). Marital status was categorized as married or not married (includes single, divorced, separated, unmarried or domestic partner, and widowed). The year of diagnosis was divided into the following intervals: 2010 to 2011 and 2012 to 2013. Histological grades was classified as grade I (well differentiated), grade II (moderately differentiated), grade III (poorly differentiated), and unknown. The primary outcome of our study was cancer-specific survival (CSS). CSS was defined as the time from the date of diagnosis to the date of either death due to ICC or last follow-up. The characteristics of the 2124 patients with ICC are described in Table 2.

**Table 2:**
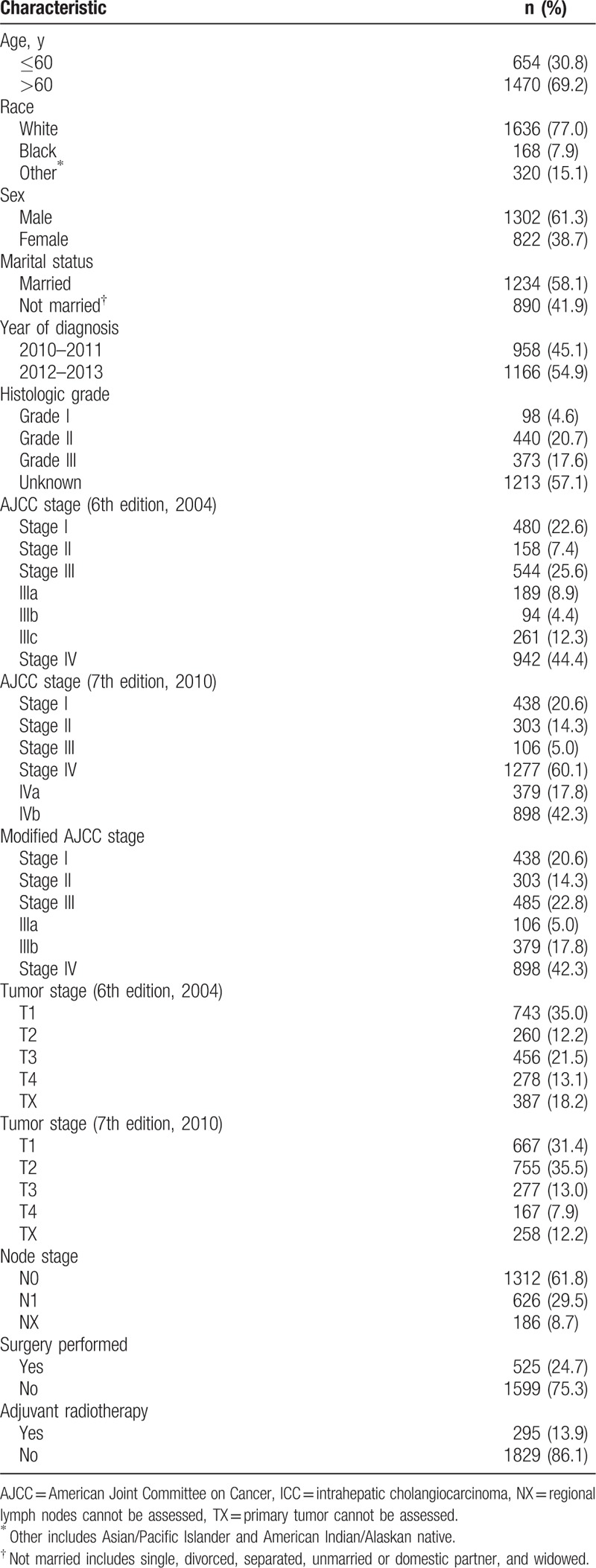
Characteristics of the 2124 patients with intrahepatic cholangiocarcinoma.

### 2.2 Statistical analysis

CSS was analyzed using Kaplan–Meier curves, and log-rank tests were used to evaluate the staging systems and other prognostic factors. Multivariate analyses for each staging system were completed using Cox proportional hazards regression models controlling for age, race, sex, marital status at diagnosis, year of diagnosis, histological grade, surgical status, and adjuvant radiotherapy status. The hazard ratio (HR) and 95% confidence interval (CI) were calculated. *P* < .05 was considered statistically significant. The prognostic performance of the different AJCC staging systems was compared in terms of homogeneity, discriminatory ability, and monotonicity. Chi-square tests of the likelihood ratio and the linear trend as well as the Akaike information criterion (AIC) were used to compare the prognostic value of the different AJCC staging systems. A Chi-square test of the likelihood ratio was used to evaluate homogeneity, a Chi-square test of the linear trend was used to evaluate the discriminatory ability and monotonicity, and the AIC was used to evaluate the relative quality of the prognostic models.^[17,18]^ Higher likelihood-ratio Chi-square and linear-trend Chi-square values indicated better homogeneity, discriminatory ability and monotonicity, and smaller AIC values indicated greater prognostic value. These computations were performed using SPSS version 13.0 for Windows (SPSS, Inc., Chicago, IL). The relative discriminative abilities of different AJCC system were assessed using the Harrell concordance index (C-index).^[19]^ A C-index value of 1.0 indicates that the model perfectly separates patients with different outcomes, and a value of 0.5 indicates that the model yields data no better than would be obtained by chance alone. The C-index was implemented by R software version 3.4.1 (http://www.r-project.org).

### 2.3 Ethics statement

For access to the SEER database, informed consent was not required, but a Data-Use Agreement for the SEER 1973–2013 Research Data File was completed.

## 3 Results

### 3.1 Patient characteristics

In total, 2124 patients from the SEER database with pathologically or clinically confirmed ICC were included in this study (Table 2). The median age at diagnosis was 66.0 years (range: 15–99 years), and the median survival time was 12.2 months (range: 0–47 months). The 1- and 3-year CSS rates were 49.2% and 26.0%, respectively. A total of 1071 patients (50.4%) died during the follow-up period. At the last follow-up date, 1053 patients (49.6%) were alive.

### 3.2 Multivariate Cox survival analyses of patients with ICC

As the AJCC staging system is predominantly composed of tumor and node stages, we did not include tumor- and node-stage variables in the multivariate Cox model because of their obvious correlation. The results indicated that AJCC stage (6th edition), AJCC stage (7th edition), and modified AJCC stage were significantly associated with CSS. In addition, we found that older age, male sex, and poor tumor differentiation were negative prognostic factors and that surgical status and adjuvant radiotherapy status were positive prognostic factors for CSS in patients with ICC (Table 3).

**Table 3:**
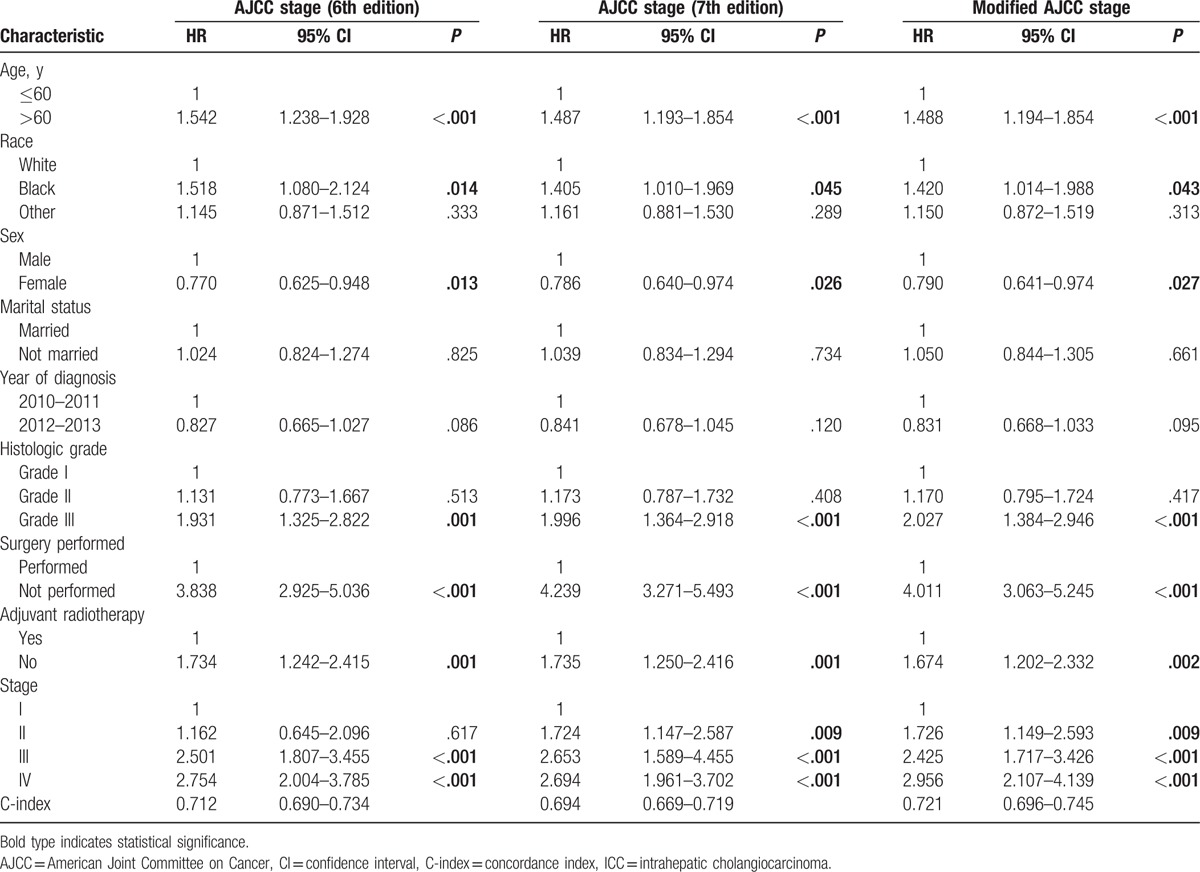
Stratified multivariate analysis of prognostic factors influencing survival in patients with intrahepatic cholangiocarcinoma.

### 3.3 AJCC staging (6th edition, 2004) classification and survival

**Figure 2:**
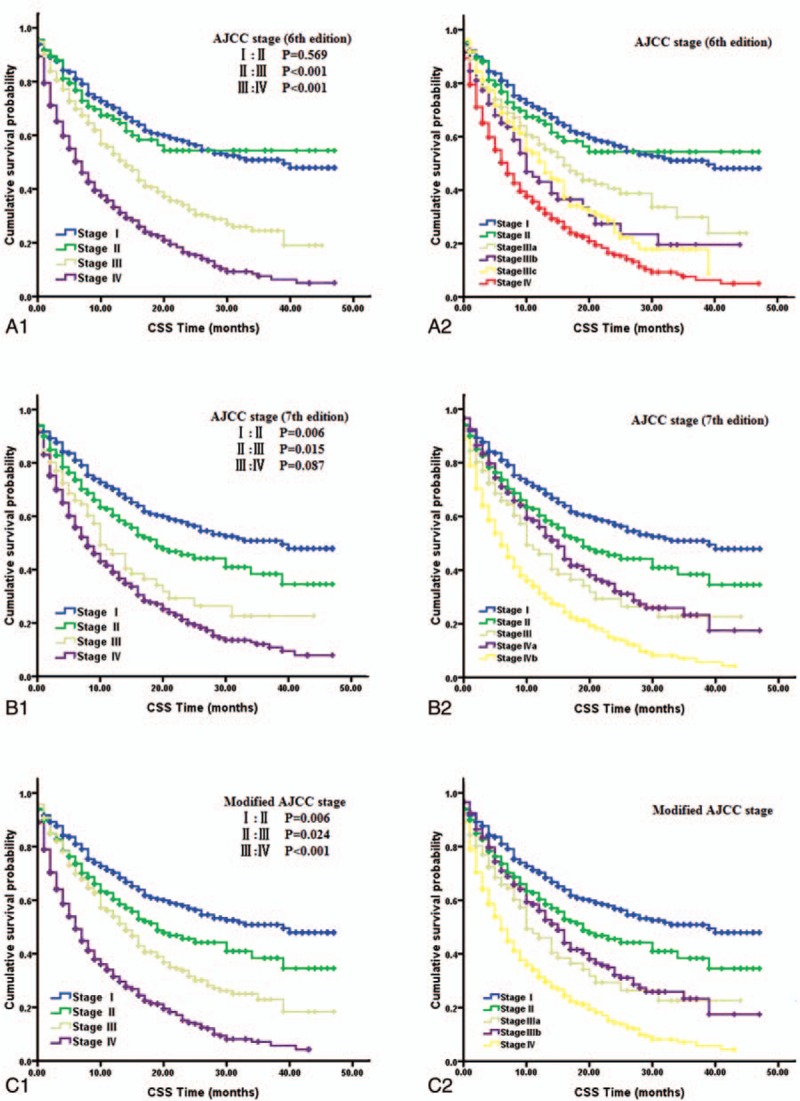
Kaplan–Meier curves of different staging classifications for patients with ICC from the SEER database. The 6th edition AJCC Staging classification (A1 and A2); the 7th edition AJCC Staging classification (B1 and B2); the modified AJCC Staging classification (C1 and C2). Overlap existed for the 6th edition AJCC staging classification of stage I and II disease (*P* = .569, A1) and of stage IIIb and IIIc disease (*P* = .512, A2). Overlap existed for the 7th AJCC staging classification of stage III and IVa disease (*P* = .169, B2), which led to a similar prognosis between stage III and IV disease (*P* = .087, B1). Survival curves were well separated by stage using the modified AJCC classification (C1 and C2). AJCC = American Joint Committee on Cancer, ICC = intrahepatic cholangiocarcinoma, SEER = Surveillance, Epidemiology, and End Results.

It is notable that the 6th edition AJCC staging classification overlapped between stage I and II disease and between stage IIIB and IIIC disease (Fig. 2, panels A1 and A2). No significant difference was observed between the HRs for stage I and stage II disease in the stratified multivariable model (with stage I as the reference value: HR for stage II, 1.162; *P* = .617; Table 3).

### 3.4 AJCC staging (7th edition, 2010) classification and survival

Using the 7th edition AJCC staging classification, only 5.0% of patients (106 of 2124) had stage III tumors (Table 2). In addition, overlap was observed in the AJCC classification of stage III and IVa disease (Fig. 2, panels B1 and B2). Relative to stage I disease, the HR for stage III disease was comparable to that of stage IV disease (stage III and IV HRs, 2.653 and 2.694, respectively; Table 3) in the stratified multivariable model.

### 3.5 Modified AJCC staging classification and survival

In consideration of the previously described shortcomings of the 6th and 7th edition AJCC staging systems, a modified AJCC staging classification was proposed; this classification system maintained the T, N, and M definitions of the 7th edition AJCC system and also adopted the AJCC 6th edition staging definitions (Table 1). A cross-tabulation of the stage distributions of all 2124 patients is presented in Table 1. Using the modified AJCC staging system, the percentage of patients with stage III disease was higher than that identified using the AJCC 7th edition system (22.8% vs 5.0%, Table 2). A substantial difference was identified in the survival curves for the different stages using the modified AJCC staging classification (Fig. 2, panels C1 and C2). In addition, a statistically significant increase in the calculated HRs with increasing disease stage was observed using the modified AJCC staging classification system (Table 3).

### 3.6 Prognostic performance of different AJCC staging systems

Chi-square tests of likelihood ratios and linear trends and AIC analysis were performed to compare the prognostic performance of the different AJCC staging systems. The results of all three tests suggested that the modified AJCC staging system had greater prognostic value than the AJCC 6th and 7th edition staging systems (Table 4). The C-index of the modified AJCC staging system was 0.721 (95% CI: 0.696–0.745), which was significantly higher than the AJCC 7th edition staging system (0.694, 95% CI: 0.669–0.719, *P* < .001), and the AJCC 6th edition staging system (0.712, 95% CI: 0.690–0.734, *P* = .033) (Table 3). These results indicated that the modified AJCC staging system was effective in predicting and discriminating the prognosis of patients with ICC.

**Table 4:**
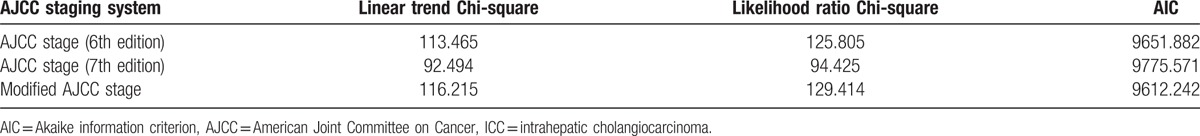
Comparison of the prognostic performance of different AJCC staging systems in patients with intrahepatic cholangiocarcinoma.

### 3.7 Comparison (*P*-value) of adjacent stages of disease using the log-rank test on stratified data

We used the log-rank test to compare prognoses among adjacent stages of disease assigned using the different AJCC staging systems from stratified data (Table 5). Statistical significance was observed only for the 6th edition AJCC staging classification of stage I and II disease and the 7th edition AJCC staging classification of stage III and IV disease using the stratified data; however, statistically significant differences were observed among all stages of disease when assigned using the modified AJCC staging classification. These differences were especially apparent for patients over 60 years in age, white patients and patients who underwent surgery.

**Table 5:**
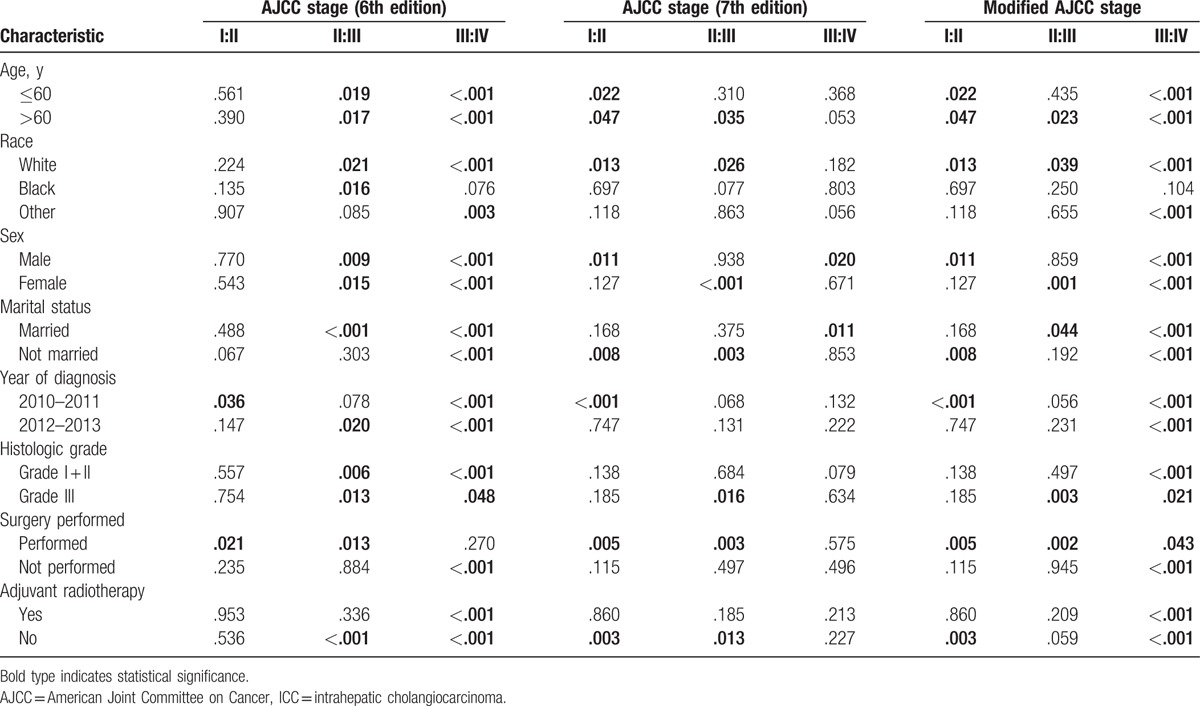
Comparison of (*P*-value) adjacent stages identified using different AJCC staging systems and log-rank tests based on stratified data.

## 4 Discussion

The incidence of ICC is increasing, and the prognosis of ICC patients remains unfavorable. Increasing numbers of therapeutic methods have been employed to target ICC, and while comprehensive therapy including surgical resection is often selected for the treatment of patients with ICC, 5-year survival ranges from only 20% to 40% in these patients,^[9]^ with a median survival time of 15 months.^[20]^ In this study, the median survival time was only 12.2 months in the group of 2124 patients with ICC obtained from the SEER database.

Based on the results of this study, we found that AJCC stage (6th edition), AJCC stage (7th edition), and modified AJCC stage variables were significantly associated with CSS in multivariate analyses. Other factors significantly associated with poor prognosis included older age, male sex, and poor tumor differentiation (Table 3). A previous meta-analysis of 7 large studies revealed that male sex, older age, poor tumor differentiation, the presence of multiple tumors, larger tumor size, lymph node metastasis, and vascular invasion were factors associated with poor prognosis in ICC patients.^[21]^ These data are similar to our results. In addition, surgical status and adjuvant radiotherapy status were significant prognostic factors related to CSS in patients with ICC; these findings were previously reported in many other studies.^[22–24]^

For the first time, ICC has been classified using a staging system independent from the AJCC staging manual.^[8]^ The 7th edition staging system is less complex than the 6th edition AJCC staging manuals^[10]^; however, the 7th edition AJCC staging system must be further validated using comprehensive and risk-stratified data rather than operative data only.^[11–14]^ In this study, we compared the AJCC 6th and 7th edition staging systems using data from 2124 patients with ICC from the SEER database. The low proportion (5.0%) of patients with stage III disease identified in this study (Table 1) suggests that the AJCC 7th edition staging classification is not ideal for the staging of ICC. In addition, relative to stage I disease, the HR for stage III disease was comparable to that for stage IV disease (stage III and IV HRs, 2.653 and 2.694, respectively; Table 3), and results from the log-rank test indicated that the AJCC classifications of stage III and IV disease did not significantly differ (*P* = .087, Fig. 2, panel B1) due to the overlap observed between stage III and IVa disease (*P* = .169, Fig. 2, panel B2). For the AJCC 6th edition staging system, our study confirmed that patients with stage I and II disease had similar prognoses (with stage I as the reference, HR for stage II, 1.162; *P* = .617; Table 3), and results from the log-rank test indicated no statistically significant difference in the AJCC classification between stage I and II disease (*P* = .569, Fig. 2, panel A1). In addition, overlap was observed between stage IIIB and IIIC disease (*P* = .512, Fig. 2, panel A2). These findings illustrate the shortcomings of the AJCC 6th and 7th edition staging systems and suggest that modifications should be made accordingly. The 7th edition staging system had a more accurate distribution for ICC stages I and II but a less accurate distribution for stage III and IV disease than the AJCC 6th edition staging system. Therefore, patients with stage III and stage IVA ICC should be defined as having stage IIIA and IIIB disease, respectively, because they have similar CSS rates. This modified system, derived from the AJCC 6th edition staging definitions, more accurately depicted the distribution of patients with stage III and stage IV disease. In addition, the low proportion (5.0%) of patients with stage III disease observed using the AJCC 7th edition staging system could also be avoided. Therefore, we propose that the AJCC 7th edition staging system be modified by maintaining its T, N, and M definitions but adopting the AJCC 6th edition staging definitions (Table 1).

The modified AJCC staging system was then compared with the AJCC 6th and 7th edition staging systems using data from the SEER database. The percentage of stage III patients with ICC identified using the modified AJCC staging system was higher than that observed using the AJCC 7th edition system (22.8% vs 5.0%, *P* < .001; Table 2). In addition, the HR for death calculated using the modified AJCC staging system for patients with stage IV disease was higher than that for patients with stage III disease (with stage I as the reference, HR for stage III, 2.425; HR for stage IV, 2.956; Table 3), and log-rank test results indicated that there was a statistically significant difference in the HR for CSS between patients with stage III with IV disease (*P* < .001, Fig. 2, panel C1). However, using the AJCC 7th edition staging system with stage I disease as the reference, the HR for death for stage III patients was 2.653, and for stage IV patients, it was 2.694 (Table 3). Corresponding log-rank tests indicated that there was no statistically significant difference in CSS between patients with stage III and stage IV disease (*P* = .087, Fig. 2, panel B1). A statistically significant increase was observed in the HRs calculated using the modified AJCC staging classification (Table 3). Moreover, based on Chi-square tests of likelihood ratios and the linear trend as well as the AIC, we found that the modified AJCC staging system had better prognostic performance than the 6th and 7th AJCC staging systems (Table 4). The survival curves were also well differentiated by ICC stage when using the modified AJCC staging system (Fig. 2, panels C1 and C2). The C-index of the modified AJCC staging system in discriminating survival of patients with ICC was 0.721, which was also significantly higher than the other staging systems (Table 3). These findings suggest that the modified AJCC staging classification may be suitable for staging ICC patients and can therefore be adopted into clinical practice.

Finally, we used the log-rank test to compare the prognoses for adjacent stages of ICC between different AJCC staging systems based on stratified data (Table 5). The results showed that the 6th edition AJCC staging classification of stage I and II disease and the 7th edition AJCC staging classification of stage III and IV disease did not significantly differ when using the stratified data; these results were in accordance with those obtained when using unstratified data. Statistically significant differences were obtained between adjacent stages using the modified AJCC staging classification, especially for patients over 60 years in age, white patients and patients who underwent surgery. Statistical significance was not observed between stage III and IV disease in the 7th edition AJCC staging classification (*P* = .575) but observed in the modified AJCC staging classification (*P* = .043) for patients who underwent surgery. The main reason may be the change of T_4_N_0_M_0_. T_4_ means the periductal infiltrating type of ICC. The periductal infiltrating type of ICC demonstrates a diffuse and often ill-defined longitudinal growth pattern along the bile duct. Uno et al^[25]^ found that the percentage of intrahepatic metastases in the periductal infiltrating type of ICC patients was significantly lower than the typical mass-forming type of ICC patients and that surgery could provide a more favorable outcome in the periductal infiltrating type of ICC patients. When we changed the place of T_4_N_0_M_0_ from the stage IV in the 7th edition AJCC staging classification to the stage III in the modified AJCC staging classification, it leads the modified AJCC staging system more suitable for staging the surgical patients. Moreover, the 8th edition is already released and will be implemented in January 1, 2018.^[26]^ But there were no reports on the advantage and applicability of this staging system. After comparing the 8th edition AJCC staging classification with our modified staging classification, we found that it was similar between them except the T_4_N_0_M_0_. In the 8th edition staging system, the T_4_ category, which described the periductal infiltrating type of ICC, was eliminated due to the controversial prognostic value,^[27–30]^ but is still recommended for data collection. In the modified staging classification, we changed the site of T_4_N_0_M_0_ from stage IV to stage III instead of eliminating it. After that, statistically significant differences were obtained between stage III and stage IV when using the modified AJCC staging classification. Therefore, before coming into effect of the 8th edition staging classification, our modified AJCC staging classification evaluated the practicability of AJCC 8th edition staging classification by using a large data set and also provided data foundation for the possible stage of periductal infiltrating type patients instead of eliminating it.

The present study had several limitations. The major problem is that we used a retrospective design and did not include tumor morphologic review. Second, the surgical method was not detailed, the radiological dose of interventional radiology was inconsistent, and other treatments (e.g., targeted therapies) were not recorded. Thus, our results may be vulnerable to confounding errors and bias.

This study is the first to propose a modified AJCC classification system. When compared with previous AJCC editions, the modified system more accurately predicted the rate of CSS in patients both overall and when stratified by risk factors. However, this study was limited by its retrospective nature, and the results need to be confirmed by prospective studies.

## References

[1] West J Wood H Logan RF Trends in the incidence of primary liver and biliary tract cancers in England and Wales 1971–2001. Br J Cancer 2006;94:1751–8. 16736026 10.1038/sj.bjc.6603127 PMC2361300

[2] Welzel TM McGlynn KA Hsing AW Impact of classification of hilar cholangiocarcinomas (Klatskin tumors) on the incidence of intra- and extrahepatic cholangiocarcinoma in the United States. J Natl Cancer Inst 2006;98:873–5. 16788161 10.1093/jnci/djj234

[3] Bergquist A von Seth E Epidemiology of cholangiocarcinoma. Best Pract Res Clin Gastroenterol 2015;29:221–32. 25966423 10.1016/j.bpg.2015.02.003

[4] Zhang H Yang T Wu M Intrahepatic cholangiocarcinoma: epidemiology, risk factors, diagnosis and surgical management. Cancer Lett 2016;379:198–205. 26409434 10.1016/j.canlet.2015.09.008

[5] Meng ZW Han SH Zhu JH Risk factors for cholangiocarcinoma after initial hepatectomy for intrahepatic stones. World J Surg 2017;41:835–43. 27766397 10.1007/s00268-016-3752-2

[6] Greene FL, American Joint Committee on Cancer, American Cancer Society. AJCC Cancer Staging Manual. 6th ed. New York: Springer-Verlag 2002.

[7] Inoue K Makuuchi M Takayama T Long-term survival and prognostic factors in the surgical treatment of mass-forming type cholangiocarcinoma. Surgery 2000;127:498–505. 10819057 10.1067/msy.2000.104673

[8] Edge SB American Joint Committee on Cancer; ACS. AJCC Cancer Staging Manual. 7th ed. New York: Springer; 2009.

[9] Nathan H Aloia TA Vauthey JN A proposed staging system for intrahepatic cholangiocarcinoma. Ann Surg Oncol 2009;16:14–22. 18987916 10.1245/s10434-008-0180-z

[10] Nathan H Pawlik TM Staging of intrahepatic cholangiocarcinoma. Curr Opin Gastroenterol 2010;26:269–73. 20179593 10.1097/MOG.0b013e328337c899

[11] Jiang W Zeng ZC Tang ZY A prognostic scoring system based on clinical features of intrahepatic cholangiocarcinoma: the Fudan score. Ann Oncol 2011;22:1644–52. 21212156 10.1093/annonc/mdq650

[12] Farges O Fuks D Le Treut YP AJCC 7th edition of TNM staging accurately discriminates outcomes of patients with resectable intrahepatic cholangiocarcinoma: by the AFC-IHCC-2009 study group. Cancer 2011;117:2170–7. 21523730 10.1002/cncr.25712

[13] Li T Qin LX Zhou J Staging, prognostic factors and adjuvant therapy of intrahepatic cholangiocarcinoma after curative resection. Liver Int 2014;34:953–60. 24134199 10.1111/liv.12364

[14] Ali SM Clark CJ Mounajjed T Model to predict survival after surgical resection of intrahepatic cholangiocarcinoma: the Mayo Clinic experience. HPB 2015;17:244–50. 25410716 10.1111/hpb.12333 PMC4333786

[15] National Cancer Institute. SEER: Surveillance, Epidemiology, and End Results Program. Available at: http://seer.cancer.gov. Accessed November, 2014.

[16] Fritz A Percy C Jack A International Classification of Diseases for Oncology. 3rd ed. Geneva, Switzerland: World Health Organization; 2000.

[17] Cho YK Chung JW Kim JK Comparison of 7 staging systems for patients with hepatocellular carcinoma undergoing transarterial chemoembolization. Cancer 2008;112:352–61. 18008352 10.1002/cncr.23185

[18] Yoon HM Ryu KW Nam BH Is the new seventh AJCC/UICC staging system appropriate for patients with gastric cancer? J Am Coll Surg 2012;214:88–96. 22036661 10.1016/j.jamcollsurg.2011.09.018

[19] Harrell FE Jr Lee KL Mark DB Multivariable prognostic models: issues in developing models, evaluating assumptions and adequacy, and measuring and reducing errors. Stat Med 1996;15:361–87. 8668867 10.1002/(SICI)1097-0258(19960229)15:4<361::AID-SIM168>3.0.CO;2-4

[20] de Jong MC Nathan H Sotiropoulos GC Intrahepatic cholangiocarcinoma: an international multi-institutional analysis of prognostic factors and lymph node assessment. J Clin Oncol 2011;29:3140–5. 21730269 10.1200/JCO.2011.35.6519

[21] Mavros MN Economopoulos KP Alexiou VG Treatment and prognosis for patients with intrahepatic cholangiocarcinoma: systematic review and meta-analysis. JAMA Surg 2014;149:565–74. 24718873 10.1001/jamasurg.2013.5137

[22] Luo X Yuan L Wang Y Survival outcomes and prognostic factors of surgical therapy for all potentially resectable intrahepatic cholangiocarcinoma: a large single-center cohort study. J Gastrointest Surg 2014;18:562–72. 24395070 10.1007/s11605-013-2447-3

[23] Hammad AY Berger NG Eastwood D Is radiotherapy warranted following intrahepatic cholangiocarcinoma resection? The impact of surgical margins and lymph node status on survival. Ann Surg Oncol 2016;23 (suppl 5):912–20. 27654107 10.1245/s10434-016-5560-1

[24] Mahadevan A Dagoglu N Mancias J Stereotactic body radiotherapy (SBRT) for intrahepatic and hilar cholangiocarcinoma. J Cancer 2015;6:1099–104. 26516357 10.7150/jca.13032 PMC4615345

[25] Uno M Shimada K Yamamoto Y Periductal infiltrating type of intrahepatic cholangiocarcinoma: a rare macroscopic type without any apparent mass. Surg Today 2012;42:1189–94. 22350300 10.1007/s00595-012-0145-5

[26] Amin MB Greene FL Edge SB The Eighth Edition AJCC Cancer Staging Manual: continuing to build a bridge from a population-based to a more “personalized” approach to cancer staging. CA Cancer J Clin 2017;67:93–9. 28094848 10.3322/caac.21388

[27] Yamasaki S Intrahepatic cholangiocarcinoma: macroscopic type and stage classification. J Hepatobiliary Pancreat Surg 2003;10:288–91. 14598147 10.1007/s00534-002-0732-8

[28] Hirohashi K Uenishi T Kubo S Macroscopic types of intrahepatic cholangiocarcinoma: clinicopathologic features and surgical outcomes. Hepatogastroenterology 2002;49:326–9. 11995443

[29] Sanada Y Kawashita Y Okada S Review to better understand the macroscopic subtypes and histogenesis of intrahepatic cholangiocarcinoma. World J Gastrointest Pathophysiol 2014;5:188–99. 25133021 10.4291/wjgp.v5.i3.188 PMC4133518

[30] Ariizumi S Yamamoto M [Surgical treatment of intrahepatic cholangiocarcinoma based on the macroscopic subtype]. Nihon Shokakibyo Gakkai Zasshi 2012;109:1885–94. 23132032

